# B-Cell-Activating Factor (BAFF) Correlated with Serum Vitamin D Values—Possible Markers with a Prognostic Role in Thyroid Autoimmune Diseases

**DOI:** 10.3390/jcm14093168

**Published:** 2025-05-03

**Authors:** Șeila Musledin, Eduard Circo, Olesea Scrinic

**Affiliations:** Endocrinology Department, Faculty of Medicine, “Ovidius” University of Constanta, 900189 Constanta, Romania; eduard_circo@yahoo.com (E.C.); olesea2005@yahoo.com (O.S.)

**Keywords:** lymphocyte-activating factor (BAFF), thyroid autoimmune pathology, vitamin D

## Abstract

**Objectives:** The aim of this study was to find correlations between vitamin D deficiency and thyroid autoimmune pathology in a group of patients from Dobrogea, a non-endemic geographical area, with a high degree of sunshine. An important factor in maintaining immunological balance is the intake of an adequate level of vitamin D. Multiple studies have suggested that vitamin D deficiency is associated with a higher incidence of autoimmune diseases. Recent studies have analyzed the possible effect of this factor in promoting autoimmunity, as the serum level of BAFF often increases among patients with systemic autoimmune diseases. **Methods:** This study included 80 patients with autoimmune thyroid pathology from the Dobrogea area. The entire study group (*n* = 80) was divided according to the established diagnosis into two study groups: Group 1 included 62 patients with CAT (chronic autoimmune thyroiditis), and Group 2 included 18 patients with GD (Graves’ disease). **Results:** Vitamin D study average values of 25-OH-vitamin D found statistically significant differences between vitamin D values in the two groups (*p* = 0.018). Determination of BAFF (B-cell-activating factor) serum levels among patients with CAT and GD obtained a lower mean value of BAFF for the CAT group compared with the GD group. The evolution of BAFF serum level related to the serum levels of the antithyroid antibodies ATPO (antithyroidperoxidase) and ATG (antithyroglobulin) was also analyzed. In the patients with GD, BAFF was not correlated with the value of ATPO or ATG, but in the patients with CAT, a correlation was found between the value of BAFF and the level of ATG but not the ATPO level. **Conclusions:** This study analyzed BAFF serum levels in patients with CAT and GD. The results indicate that BAFF acts as a stimulatory factor of immunoglobulin production in autoimmune diseases. These results require clarifying the role and therapeutic benefits of supplementing vitamin D intake in patients with autoimmune diseases.

## 1. Introduction

Autoimmune diseases are characterized by changes in immune homeostasis, leading to erroneous self-antigen recognition, followed by the destruction of the involved tissue by autoreactive immune cells [1].

Not only do genetic, epidemiological [2,3] factors contribute to the occurrence of autoimmune diseases but also environmental factors. An important factor is the availability of an adequate level of vitamin D. Multiple studies suggest that vitamin D deficiency is associated with a higher incidence of autoimmune diseases [1,4,5]. Experimental and clinical data provide evidence that vitamin D is one of the environmental factors that can increase the prevalence of autoimmune diseases (systemic lupus erythematosus, rheumatoid arthritis, insulin-dependent diabetes, multiple sclerosis, and inflammatory bowel diseases) [6,7].

Autoimmunity is involved in the pathogenesis of some thyroid diseases, including Graves’ disease (GD) and chronic autoimmune (Hashimoto’s) thyroiditis (CAT/HT). BAFF (B-lymphocyte-activating factor), belonging to the tumor necrosis factor (TNF) family, is a vital cytokine for B cells that helps regulate both innate and adaptive immune responses. Elevated serum levels of BAFF are found in a number of different autoimmune diseases, and BAFF is found at inflammatory sites where there is lymphoid neogenesis. BAFF antagonism has been used in several autoimmune disease models, resulting in B cell depletion, decreased T cell and DC activation, and a reduction in the overall inflammatory burden. BAFF, through its interaction with its receptor (BAFF-R), is required for the survival of mature naïve B cells, all of which are depleted by the blockade of BAFF [8].

Being a cytokine belonging to the TNF family, BAFF is expressed by B cells, T cells, neutrophils, monocytes, dendritic cells, stromal lymphoid cells, and malignant B cells. As a result of the binding of BAFF to the specific receptor, there is a stimulation of B cells that leads to an increased production of antibodies by increasing the survival time of the B cell, and to the proliferation and blocking of autoreactive B cells. Consequently, recent studies have analyzed the possible effect of the BAFF factor in promoting autoimmunity [9]. The serum level of BAFF was also increased among patients with autoimmune diseases with varied tissue substrates such as Sjogren’s syndrome, rheumatoid arthritis, autoimmune hepatitis, and primary biliary cirrhosis [10,11].

Studies of GD and associated orbitopathy regarding the effect of BAFF on disease progression have reported higher BAFF concentrations in patients with GD compared with controls but no difference between patients with active or inactive GD. BAFF concentrations were also significantly correlated with serum ATG antibodies but not with age, sex, smoking, or treatment. Also, no correlations were found with the serum level of ATPO or TRAb (TSH receptor antibodies) [12].

Another study on autoimmune thyroid diseases in which patients with HT and GD were enrolled studied the effect of BAFF and 28 other circulating factors (IFN-α, IL-4, TNF-α, eotaxin, etc.) in the evolution of these pathologies. It was reported that BAFF was the best circulating indicator to identify GD and HT among all 29 chosen biomarkers and could be used to predict disease severity in HT and active GD. Significant associations of serum BAFF and TNF-α levels with serum free thyroxine (FT4) were found in patients with HT, and, in active GD, serum BAFF was also correlated with serum FT4 [13].

The novelty of the present study lies in the use of BAFF as a research element in autoimmune thyroid diseases, an area where the existing literature remains scarce regarding its involvement. While BAFF has been extensively studied in rheumatologic autoimmune conditions, its role in thyroid-specific autoimmunity, such as Hashimoto’s thyroiditis and Graves’ disease, has only recently begun to draw attention. Evidence suggests that elevated serum BAFF levels may contribute to autoimmune dysfunction through enhanced B cell activation and survival, thus supporting its potential role in thyroid autoimmune pathogenesis.

The association of some autoimmune diseases in certain categories of patients suggests etiopathogenic interferences, with a common lesional substrate [14].

## 2. Materials and Methods

This observational and descriptive clinical study was conducted over a period of one year (January 2021–January 2022) and included 80 patients with autoimmune thyroid disease from the Dobrogea region. The entire study group (n = 80) was divided according to the established diagnosis into two study groups: Group 1—including 62 patients (77.5%) with CAT and Group 2—including 18 patients (22.5%) with GD. Inclusion criteria comprised the presence of autoimmune thyroid disease and residency in Constanța County. Exclusion criteria included lack of informed consent, coexisting autoimmune diseases, or other unrelated pathologies. Clinical evaluation included patient history (age, sex, geographic origin, medical history, current treatment), general physical examination (weight, height, BMI calculation), and specific thyroid examination (inspection and palpation for goiter or nodules). Each patient also underwent thyroid ultrasound using a high-frequency linear transducer (7.5–12 MHz) to assess thyroid volume, echogenicity, vascularization, and any structural abnormalities. Evaluation of the patients in this study followed several stages: anamnesis, clinical examination, and paraclinical examination, which included hormone dosage—TSH, FT4, ATPO, ATG, TRAb, and 25-OH-vitamin D dosage—as well as the classification of patients according to the value obtained (severe deficiency < 10 ng/mL; deficiency: 10–20 ng/dl; insufficiency 21–29 ng/mL; optimal level > 30 ng/mL). Serum BAFF levels were analyzed for both study groups, relative to demographic factors, serum vitamin D level, and factors specific to thyroid dysfunction. BAFF serum levels were measured using the SimpleStep Human BAFF ELISA^®^ Kit (TNFSF13B, Medist Life Science, Abcam, PA, United States), an enzyme-linked immunosorbent assay (ELISA) with a reference range of 0.08–5 ng/mL and a sensitivity of 12.7 ng/mL. The technique is based on antigen–antibody binding followed by colorimetric detection. BAFF, encoded by the *TNFSF13B gene*, plays a key role in B-cell survival, differentiation, and immune regulation.

### Statistical Analysis

For the interpretation of the obtained results, the following were used: the Chi square test for non-numerical parameters and the frequency distribution being analyzed; Student’s *t*-test; linear regression—simple and multifactorial (Pearson’s r coefficient); and ANOVA test to compare the results of the two groups.

## 3. Results

The CAT group included 62 patients (50 women and 12 men), and the GD group included 18 patients (17 women and 1 man) aged between 18 and 75 years.

**Figure 1:**
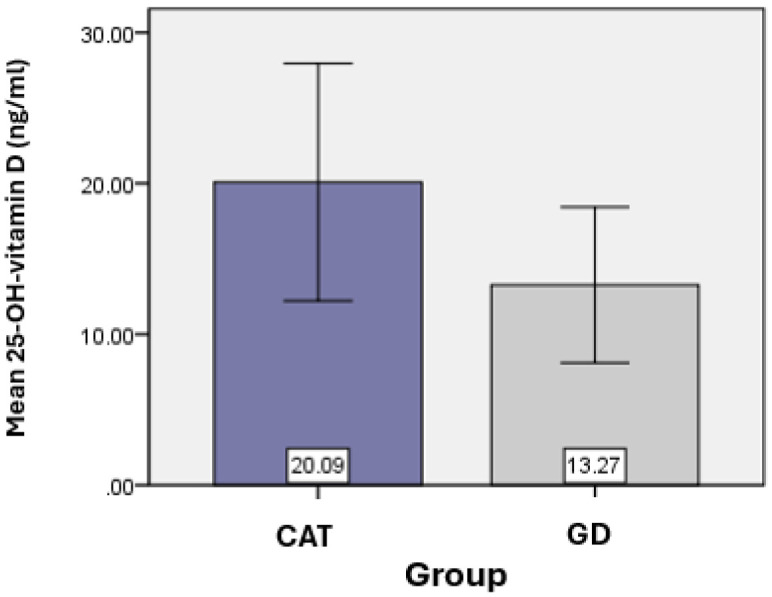
Graphical representation of the average values of vitamin D for the groups analyzed.

Vitamin D was considered an essential element of the present study. The average value of 25-OH-vitamin D obtained for the CAT group (*n* = 62) was 20.09 ± 7.87 ng/mL, and for the GD group (*n* = 18) the average value of 25-OH-vitamin D was 13.27 ± 5.16 ng/mL, with statistically significant differences found between the vitamin D values of the two groups (*p* = 0.018). (Table 1) (Figure 1).

Based on the serum values of vitamin D obtained, they were divided into categories of severity both in the group of those with CAT and those with GD (Table 2).

The analysis of the vitamin D status related to the age of the patients noted a slightly higher average value of the age of patients with hypovitaminosis D compared to the average value of the age corresponding to an optimal level both in patients with CAT and in those with GD.

**Figure 2:**
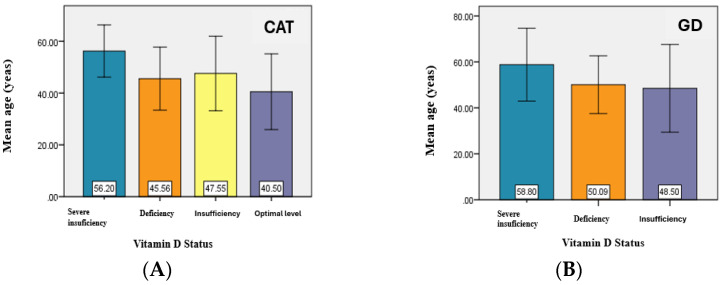
Graphical representation of the mean age values for CAT study group (**A**) and GD study groups (**B**).

There are significant differences between the average age values for at least two of the analyzed groups—corresponding to the average values for the groups—severe deficiency and deficiency, severe deficiency and insufficiency, and severe deficiency and optimal level (*p* < 0.05) (Figure 2).

The analysis of the vitamin D status by sex in the two study groups revealed that, in the CAT group, 54 patients had low vitamin D levels, of whom 44 were female and 10 were male. In the GD group, all patients exhibited low vitamin D levels, with 17 being female and 1 being male (Table 3).

Although hypovitaminosis D was more notable in female patients compared to male patients, in whom vitamin D values were slightly higher, no statistically significant association was identified between gender and vitamin D status in either the CAT group (*p* = 0.457) or the GD group (*p* = 0.714). This finding, particularly in the GD group, may be explained by an unequal gender distribution.

The evaluation of serum level of ATPO and ATG antibodies in relation to vitamin D status was also of particular interest for patients with CAT and GD.

**Figure 3:**
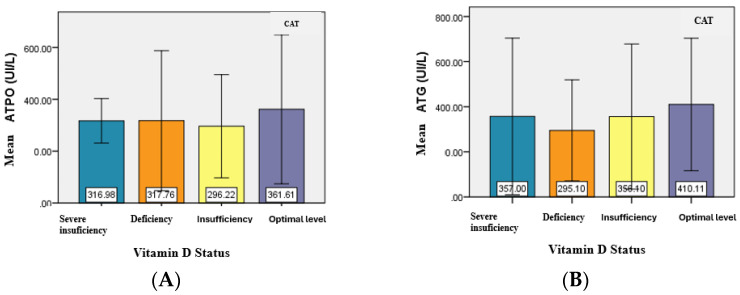
Graphical representation of the mean values of ATPO (**A**) and ATG (**B**) for CAT patients.

For patients in the CAT group, the mean serum level of ATPO did not have significant differences (*p* = 0.931) in relation to vitamin D status. However, in patients with severe vitamin D deficiency, the minimum ATPO value was significantly higher, and the maximum value was lower, compared to the corresponding values observed in the other subgroups (deficiency, insufficiency, and optimal levels) (Table 4) (Figure 3).

The analysis of the relationship between ATG levels and vitamin D status revealed no significant differences between the mean ATG values, similar to the mean values of ATPO. Although no statistically significant association was identified between the type of antibodies present and the status of vitamin D (*p* = 0.718), the majority of patients with severe deficiency, deficiency, and insufficiency had both types of positive antibodies. (Table 5).

**Figure 4:**
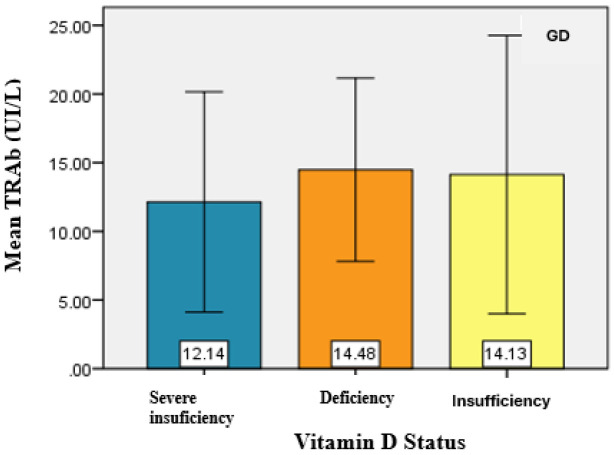
Graphical representation of the mean TRAB values for the analyzed groups.

In the GD group, TRAb served as the marker of the disease, and its mean values were analyzed for the existence of an association between the level of TRAB and vitamin D status. However, no statistically significant result was obtained between the two studied variables (Figure 4).

The determination of the lymphocyte-activating factor (BAFF) was performed with the aim of looking for potential associations between its serum concentration, variations in vitamin D deficiency, and thyroid hormone levels, and their variations, to assess the possible involvement of BAFF in amplifying the autoimmune thyroid process.

Determination of BAFF serum levels (pg/mL) among patients with CAT and GD revealed that the mean BAFF value for the CAT group was 0.12 ± 0.10 ng/mL, while in the GD group, the mean BAFF value was 0.43 ± 0.17 ng/mL. (Table 6).

**Figure 5:**
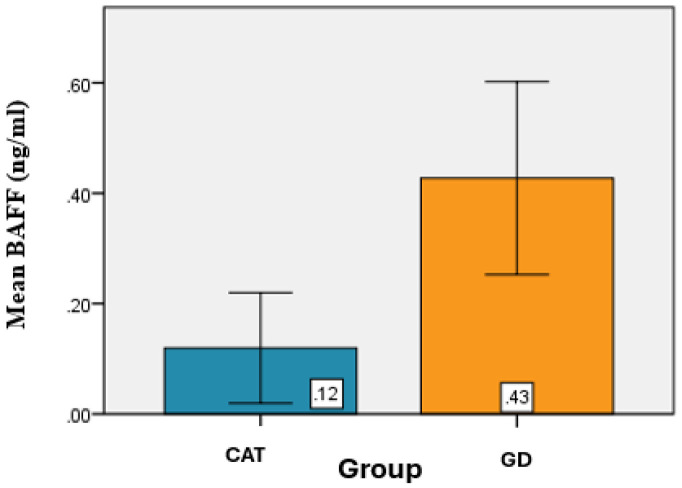
Graphical representation of the average BAFF values for the groups analyzed.

From a statistical perspective, significant differences were identified between the mean BAFF values of the CAT and GD groups (*p* < 0.001) (Figure 5).

The analysis of the serum BAFF levels between the two study groups (CAT and GD) in relation to vitamin D serum levels revealed slightly higher values of BAFF in patients with hypovitaminosis D compared to patients with CAT. Moreover, in GD patients, BAFF values were higher and appeared to be proportional to the degree of vitamin D deficiency. (Table 7).

**Figure 6:**
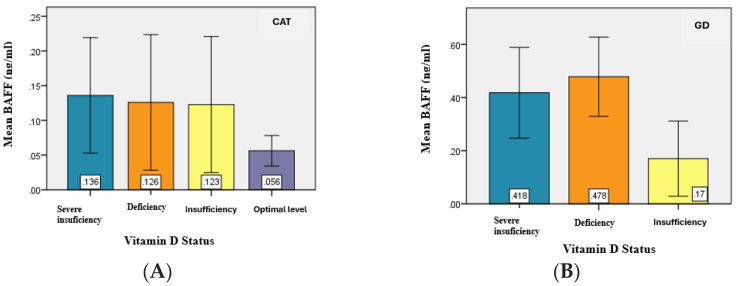
Graphical representation of mean BAFF values for the study groups. (**A**) CAT, (**B**) GD.

The statistical analysis demonstrated significant differences (*p* = 0.031) in the mean values of BAFF serum levels between at least two of the analyzed groups—specifically between patients with severe deficiency and optimal levels, deficiency and optimal levels, and insufficiency and optimal levels (*p* < α = 0.05) (Figure 6).

The analysis of BAFF serum levels according to patient age indicated that no significant correlation was observed between these two variables.

Regarding gender, in the CAT group, the mean BAFF serum level was 0.11 ± 0.09 ng/mL in female patients and 0.14 ± 0.12 ng/mL in male patients. In the GD group, the mean BAFF serum level was 0.42 ± 0.18 ng/mL for female patients and 0.56 ng/mL for the male patient (Table 8).

Furthermore, correlation analysis between BAFF and ATPO serum levels showed no significant association in either the CAT group (*p* = 0.246) or the GD group (*p* = 0.929) (Table 9).

Regarding the correlation between BAFF serum levels and ATG levels in the two study groups, the analysis revealed that, in CAT patients, a significant correlation was identified (*p* = 0.038). In contrast, no significant correlation was found between these variables in the GD group (*p* = 0.204. (Table 10).

The determination of BAFF serum levels and the type of antibodies present in the two study groups indicated that among the CAT group the minimum serum value of BAFF for patients with both ATPO and ATG was 0.01 pg/mL, while the maximum value was 0.30 ng/mL. In the GD group, the minimum value of BAFF for patients with both ATPO and ATG was 0.07 ng/mL, and the maximum value of BAFF reached 0.59 ng/mL. The analysis of BAFF serum level in the group with GD indicated a mean value of 0.39 ± 0.18 pg/mL for patients with positive ATPO, ATG, and TRAb antibodies. For patients with positive ATG and TRAb, the mean value of BAFF was 0.56 ± 0.02 pg/mL DS, whereas for those with positive ATPO and TRAb, the mean value was 0.42 pg/mL ± 0.2 DS (Table 11).

## 4. Discussion

BAFF is a protein belonging to the TNF family [15], playing an important role in regulating the recombination and selection of autoreactive B cells. Although the mechanism regulating BAFF levels is not fully understood, BAFF concentrations depend primarily on the number of B cells and the expression of BAFF binding receptors [16]. These findings raise the question of a significant difference in the “intensity” of the autoimmune process across different thyroid diseases.

In this context, the present study analyzed the serum BAFF levels in patients with CAT and GD because of the fact that BAFF acts as a stimulating factor for immunoglobulin production in autoimmune diseases.

The analysis revealed significant differences between the average BAFF levels in the CAT and GD groups (*p* < 0.001), consistent with findings reported in previous studies [17]. These findings support the hypothesis of a significant difference in the “intensity” of the autoimmune process in these two conditions.

The aim was to determine the existence of a possible association between the serum levels of 25-OH-vitamin D and the serum levels of BAFF in the two groups studied, but despite the fact that the serum levels of BAFF were significantly higher in patients with GD and hypovitaminosis D was more pronounced, no statistically significant correlation was identified between BAFF and vitamin D serum levels across the study groups.

The evaluation of BAFF serum levels according to patient age revealed slightly higher values of BAFF in patients with GD. Moreover, in this group, BAFF levels appeared to be directly proportional to patient age [17].

Determination of the BAFF serum level according to gender among the studied patients indicated that both in the CAT group and in the GD group, the average values were higher in male patients, although in the specialized literature, the BAFF value is higher among women with autoimmune thyroid diseases. A possible explanation is the fact that the estrogen levels can modulate BAFF expression, thus determining a higher incidence of autoimmune thyroid pathology among women [18].

The relationship between BAFF serum levels and the levels of antithyroid antibodies (ATPO and ATG) was also examined. 

In GD patients, BAFF levels did not correlate with either ATPO or ATG levels.

However, among CAT patients, a significant correlation was found between BAFF levels and ATG levels, but not with ATPO levels. Additionally, in GD patients, BAFF levels were analyzed in relation to TRAb levels; however, no correlation was identified. 

It should be mentioned that one possible explanation for the significantly increased BAFF values among patients with GD is that 55% of them also presented with ophthalmopathy. Data from previous studies [19] indicate significantly increased BAFF levels among patients with GD associated with ocular involvement [20].

Finally, the findings regarding BAFF levels and their association with various clinical and biochemical parameters potentially involved in the thyroid autoimmune process were heterogeneous but consistent with existing data in the literature [20,21].

## 5. Conclusions

The results of this study point to both the complexity of autoimmune activation and the individual variability in its clinical and immunological expression.

Although BAFF has been primarily investigated in the context of rheumatologic autoimmune diseases, emerging evidence highlights its potential involvement in autoimmune thyroid disorders as well. Elevated serum BAFF levels have been observed in patients with Hashimoto’s thyroiditis and Graves’ disease, suggesting a role in promoting B-cell-mediated autoimmunity.

Furthermore, the association between BAFF levels and thyroid-associated ophthalmopathy, along with variations depending on the type of immunosuppressive therapy, supports its relevance as both a biomarker and a potential therapeutic target in autoimmune thyroid pathology.

Thus, the evaluation of 25-OH-vitamin D and BAFF serum levels may contribute to a better characterization of thyroid autoimmune status with individual diagnostic, functional, prognostic, and therapeutic implications.

Further extensive studies are required to establish standardized diagnostic criteria and therapeutic principles in patients with autoimmune thyroid disease.

At the same time, it is necessary to specify the role and therapeutic benefits of supplementing the intake of vitamin D in patients with autoimmune thyroid diseases. Nonetheless, it is important to acknowledge that the present study is observational in nature, which limits the ability to establish causal relationships.

## Figures and Tables

**Table 1:** Determination of the main statistical indicators of the serum level of vitamin D, comparatively, between the two study groups.

25-(OH)-Vitamin D (ng/mL)			
		Group	
		TCA	GD
N	Valid	62	18
Mean		20.09	13.27
Median		19.10	11.65
Mode		16.00	11.00
Std. Deviation		7.87	5.16
Minimum		7.00	7.00
Maximum		39.00	28.00
Percentiles	25	14.75	9.93
	50	19.10	11.65
	75	25.00	16.25

**Table 2:** Distribution of patients from both study groups according to the vitamin D status.

			Vitamin D Status				Total
			Severe Deficiency	Deficiency	Insufficiency	Optimal Level	
Group	CAT	Count	5	27	22	8	62
		% of Total	6.3%	33.8%	27.5%	10.0%	77.5%
	GD	Count	5	11	2	0	18
		% of Total	6.3%	13.8%	2.5%	0.0%	22.5%
Total		Count	10	38	24	8	80
		% of Total	12.5%	47.5%	30.0%	10.0%	100.0%

**Table 3:** Sex-based distribution of vitamin D status among CAT and GD groups.

			Gender		Total
			F	M	
Vitamin D Status CAT	Severe deficiency	Count	5	0	5
		% of Total	8.1%	0.0%	8.1%
	Deficiency	Count	23	4	27
		% of Total	37.1%	6.5%	43.5%
	Insufficiency	Count	16	6	22
		% of Total	25.8%	9.7%	35.5%
	Optimal level	Count	6	2	8
		% of Total	9.7%	3.2%	12.9%
Total		Count	50	12	62
		% of Total	80.6%	19.4%	100.0%
Vitamin D Status GD	Severe deficiency	Count	5	0	5
		% of Total	27.8%	0.0%	27.8%
	Deficiency	Count	10	1	11
		% of Total	55.6%	5.6%	61.1%
	Insufficiency	Count	2	0	2
		% of Total	11.1%	0.0%	11.1%
Total		Count	17	1	18
		% of Total	94.4%	5.6%	100.0%

**Table 4:** Main statistical indicators for ATPO and ATG values in relation to vitamin D status in the CAT group.

**ATPO (UI/mL)**					
	**N**	**Mean**	**Standard Deviation**	**Minimum**	**Maximum**
Severe deficiency	5	316.98	85.86	229.00	410.00
Deficiency	27	317.76	270.02	11.00	850.00
Insufficiency	22	296.22	198.98	15.00	600.00
Optimal level	8	361.61	287.53	23.00	875.00
**ATG (UI/mL)**					
	**N**	**Mean**	**Standard Deviation**	**Minimum**	**Maximum**
Severe deficiency	5	357.00	347.30	20.00	790.00
Deficiency	27	295.10	224.37	17.00	898.00
Insufficiency	22	356.10	321.80	15.00	890.00
Optimal level	8	410.11	293.93	23.00	879.00

**Table 5:** Type of antibodies present among CAT patients in relation to vitamin D status.

			ATPO/ATG/ATPO + ATG			Total
			ATPO+	ATG+	ATPO/ATG+	
Vitamin D Status	Severe deficiency	Count	2	0	3	5
		% of Total	3.2%	0.0%	4.8%	8.1%
	Deficiency	Count	5	3	19	27
		% of Total	8.1%	4.8%	30.6%	43.5%
	Insufficiency	Count	8	2	12	22
		% of Total	12.9%	3.2%	19.4%	35.5%
	Optimal level	Count	1	1	6	8
		% of Total	1.6%	1.6%	9.7%	12.9%
Total		Count	16	6	40	62
		% of Total	25.8%	9.7%	64.5%	100.0%

**Table 6:** Main statistical indicators of BAFF levels in the two study groups.

BAFF (pg/mL)			
		Group	
		CAT	GD
N	Valid	62	18
Mean		0.12	0.43
Median		0.09	0.49
Mode		0.04	0.56
Std. Deviation		0.09	0.17
Minimum		0.01	0.07
Maximum		0.40	0.59
Percentiles	25	0.05	0.34
	50	0.09	0.49
	75	0.15	0.56

**Table 7:** BAFF serum levels (ng/mL) among study patients according to vitamin D status.

BAFF (pg/mL)						
Group		N	Mean	Standard Deviation	Minimum	Maximum
TCA	Severe deficiency	5	0.14	0.08	0.04	0.20
	Deficiency	27	0.13	0.10	0.02	0.40
	Insufficiency	22	0.12	0.10	0.01	0.40
	Optimal level	8	0.06	0.02	0.03	0.10
BG	Severe deficiency	5	0.42	0.17	0.12	0.55
	Deficiency	11	0.48	0.15	0.09	0.59
	Insufficiency	2	0.17	0.14	0.07	0.27

**Table 8:** Correlation between age and BAFF serum levels within the two groups.

Groups			Age (Years)
CAT	BAFF (pg/mL)	Pearson Correlation	0.214
		Sig. (2-tailed)	0.094
		N	62
GD	BAFF (pg/mL)	Pearson Correlation	0.059
		Sig. (2-tailed)	0.816
		N	18

**Table 9:** Correlations between BAFF and ATPO levels in the two study groups.

Group			ATPO (UI/mL)
CAT	BAFF (pg/mL)	Pearson Correlation	−0.150
		Sig. (2-tailed)	0.246
		N	62
GD	BAFF (pg/mL)	Pearson Correlation	0.023
		Sig. (2-tailed)	0.929
		N	18

**Table 10:** Correlation between BAFF and ATG levels in the two study groups. * Indicates that this correlation is statistically significant.

Group			ATG (UI/mL)
CAT	BAFF (pg/mL)	Pearson Correlation	−0.264 *
		Sig. (2-tailed)	0.038
		N	62
GD	BAFF (pg/mL)	Pearson Correlation	0.314
		Sig. (2-tailed)	0.204
		N	18

**Table 11:** Distribution of patients with GD according to the type of antibodies and BAFF levels.

ATPO/ATG/ATPO+ATG		N	Minimum	Maximum	Mean	Standard Deviation
ATPO+	BAFF (pg/mL)	4	0.12	0.54	0.42	0.20
ATG+	BAFF (pg/mL)	3	0.55	0.58	0.56	0.02
ATPO/ATG+	BAFF (pg/mL)	11	0.07	0.59	0.39	0.18

## Data Availability

The original contributions presented in this study are included in the article. Further inquiries can be directed to the corresponding author.

## Abbreviations

The following abbreviations are used in this manuscript:

CAT	Chronic autoimmune thyroiditis
GD	Graves’ disease
BAFF	B-cell-activating factor
ATPO	Antithyroid peroxidase
ATG	Antithyroglobulin antibody
TRAb	TSH-R antibody
